# Projection of Damaged Visual and Language Regions on Low Trail Making Test Part-B Performance in Stroke Patients

**DOI:** 10.3389/fneur.2022.853942

**Published:** 2022-06-02

**Authors:** Ayako Nishimura, Stephanie Sutoko, Masashi Kiguchi, Hirokazu Atsumori, Akiko Obata, Tsukasa Funane, Akihiko Kandori, Tomohiko Mizuguchi, Koji Shimonaga, Seiji Hama, Toshio Tsuji

**Affiliations:** ^1^Center for Exploratory Research, Research & Development Group, Hitachi. Ltd., Kokubunji, Japan; ^2^Department of Rehabilitation, Hibino Hospital, Hiroshima, Japan; ^3^IoT Innovation Department, New Business Produce Division, Maxell Ltd., Yokohama, Japan; ^4^Department of Neurosurgery and Interventional Neuroradiology, Hiroshima City Asa Citizens Hospital, Hiroshima, Japan; ^5^Department of Neurosurgery, Graduate School of Biomedical and Health Sciences, Hiroshima University, Hiroshima, Japan; ^6^Graduate School of Advanced Science and Engineering, Hiroshima University, Higashihiroshima, Japan

**Keywords:** data-driven classification, trail making test part-B, brain lesions, stroke patients, AAL atlas, unsupervised *k*-means clustering

## Abstract

**Background:**

The Trail Making Test Part-B (TMT-B) is an attention functional test to investigate cognitive dysfunction. It requires the ability to recognize not only numbers but also letters. We analyzed the relationship between brain lesions in stroke patients and their TMT-B performance.

**Methods:**

From the TMT-B, two parameters (score and completion time) were obtained. The subjects were classified into several relevant groups by their scores and completion times through a data-driven analysis (*k*-means clustering). The score-classified groups were characterized by low (≤10), moderate (10 < score < 25), and high (25) scores. In terms of the completion time, the subjects were classified into four groups. The lesion degree in the brain was calculated for each of the 116 regions classified by automated anatomical labeling (AAL). For each group, brain sites with a significant difference (corrected *p* < 0.1) between each of the 116 regions were determined by a Wilcoxon Rank–Sum significant difference test.

**Results:**

Lesions at the cuneus and the superior occipital gyrus, which are mostly involved in visual processing, were significant (corrected *p* < 0.1) in the low-score group. Furthermore, the moderate-score group showed more-severe lesion degrees (corrected *p* < 0.05) in the regions responsible for the linguistic functions, such as the superior temporal gyrus and the supramarginal gyrus. As for the completion times, lesions in the calcarine, the cuneus, and related regions were significant (corrected *p* < 0.1) in the fastest group as compared to the slowest group. These regions are also involved in visual processing.

**Conclusion:**

The TMT-B results revealed that the subjects in the low-score group or the slowest- group mainly had damage in the visual area, whereas the subjects in the moderate-score group mainly had damage in the language area. These results suggest the potential utility of TMT-B performance in the lesion site.

## Introduction

The Trail Making Test (TMT) was developed as a neuropsychological test that is commonly used to evaluate higher-order attention functions (1–4). The TMT has two parts, Part A and Part B. The Trail Making Test Part-A (TMT-A) displays numbers at random points, and the subject is asked to connect the numbers in ascending order by making a “trail.” In Trail Making Test Part-B (TMT-B), the subjects are required to alternately connect both numbers and letters (hiragana script in the Japanese version) in ascending order (5). The TMT-B is thus more difficult than the TMT-A. The TMT requires capabilities such as number and character recognition, attention persistence and force, and, movement of the hand during visual search. Accordingly, it is widely used for evaluating acquired brain dysfunction and cognitive dysfunction.

When the brain is damaged by stroke, various types of higher brain (e.g., cognitive) dysfunction and mood disorders (e.g., depression) develop according to the damaged site in the brain. As a result, the analysis of brain sites concerning higher brain dysfunction and depression is an active research topic. For example, the type of attentional deficit after stroke has been reported to depend on the lesion site (6). Furthermore, a method has been developed to use the degree of damage in each brain site to classify patient groups through data-driven analysis and related methods. By using this method, the brain site related to depression has been identified from depression-related indicators as the right Rolandic operculum (7, 8). Analysis of the relationship between the brain site and test results or mood-related indicators has also advanced (7, 9).

**Figure 1:**
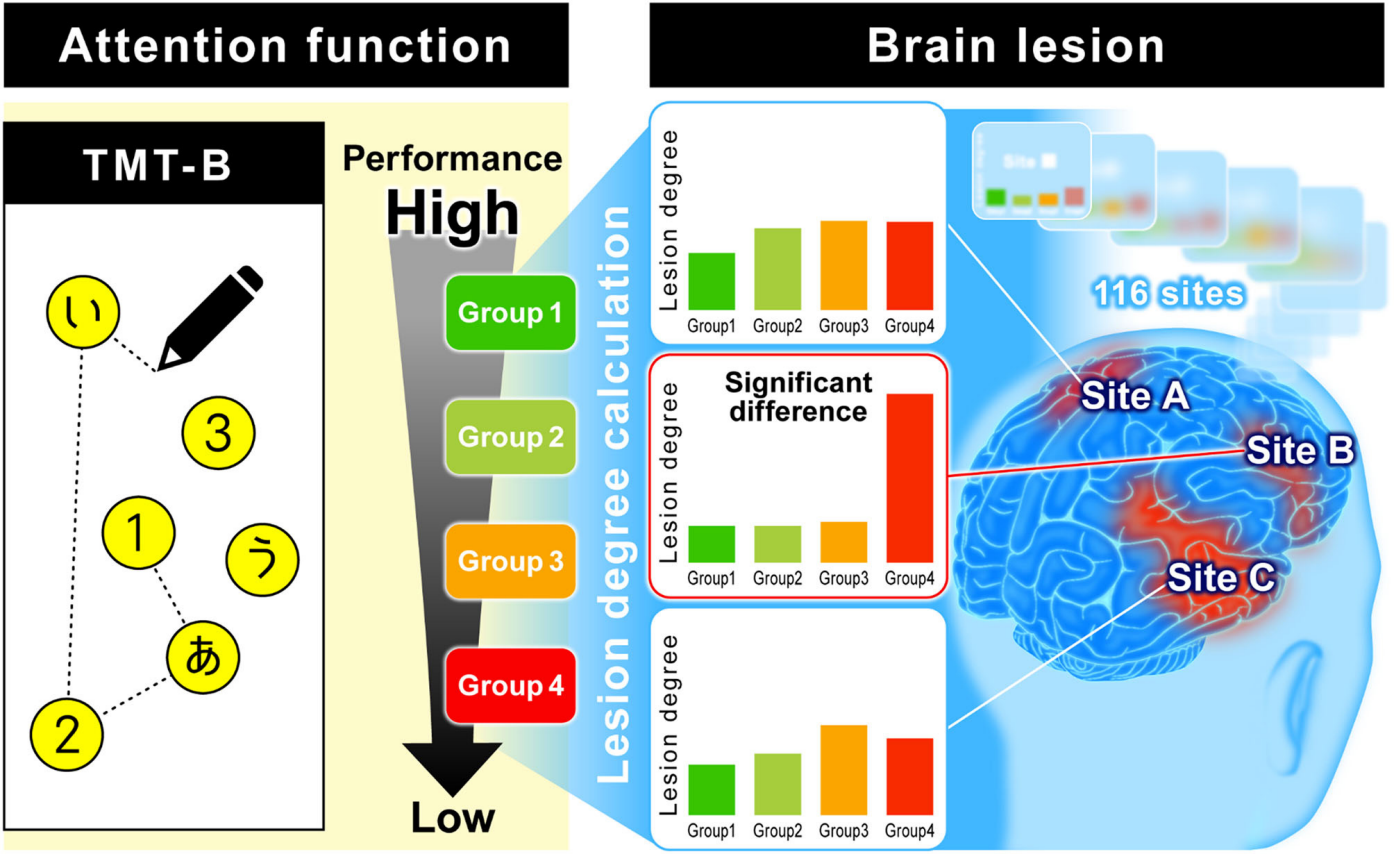
Analysis of relationship between TMT-B performance and brain lesion. Subjects were classified into several relevant groups by TMT-B performance using a data-driven analysis. The brain is divided into 116 regions classified by AAL. The total number of lesions and voxels for each region was calculated, and the ratio of lesion volume was calculated as “lesion degree (%).” We explore where there is a difference in lesion degree among groups.

In addition to assessing attention persistence, visual search force, and hand movement, the TMT-B includes a language task, as it requires recognizing numbers and characters. Actually, many stroke patients can complete TMT-A, while some patients cannot reach the end of TMT-B. Hence, we investigated the relationship between TMT-B results and lesion sites observed using MRI to find the dominant functions affecting the TMT-B results. In this study, to analyze the relationship between brain lesions in stroke patients and TMT-B performance, we used a previously developed method that is illustrated in Figure 1 (8). For the TMT-B, the completion time to connect all 25 points is generally used as the test score; however, stroke patients often cannot complete all 25 points. Accordingly, we defined the number of points that a subject could connect correctly as the TMT-B score, in addition to the completion time.

## Methods

### Subjects

**Figure 2:**
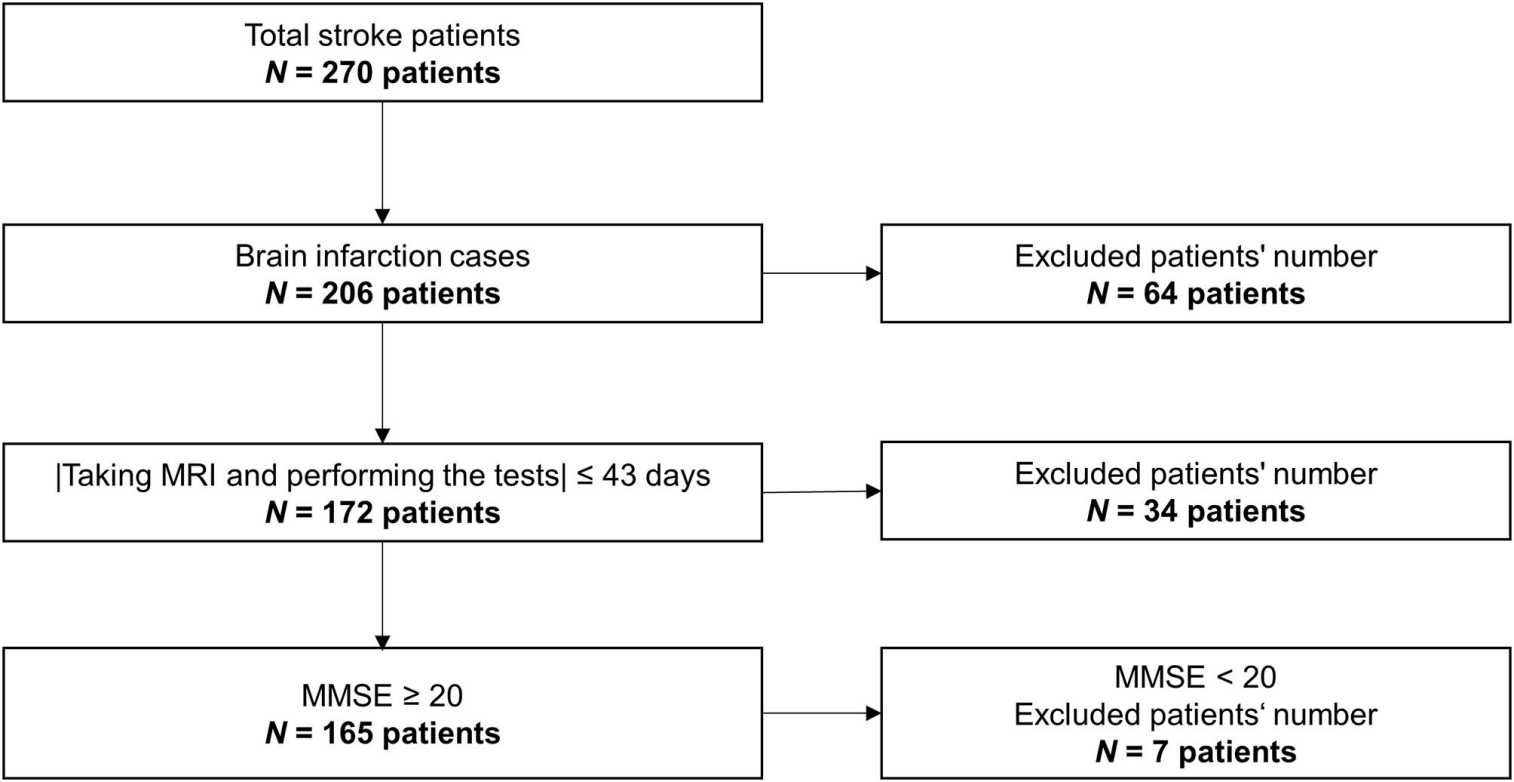
Flowchart for selecting patients' data.

The data used here were obtained from a retrospective study. The data were collected from 270 stroke patients in the rehabilitation ward of Hibino Hospital in Hiroshima, Japan. The data included medical records, MRI brain-image data, and data from the higher brain-function tests, namely, the TMT and the Mini-Mental State Examination (MMSE). Figure 2 shows a flowchart of how the patients' data were selected. Only brain infarction cases were analyzed in this study with 206 patients. After considering the effects of the measurement intervals, we excluded 34 patients who did not meet the condition that the time interval between taking the MRI and performing the tests had to be 43 days or less. Even though all patients could understand the test instructions, seven patients with a low MMSE score (below 20) were excluded because of the possibility of an increased risk of aphasia. As a result, the data from a total of 165 patients (134 males; 66.8 ± 9.6 years old; 39–86 years old) were used in this study. Table 1 shows patient information. Means and standard deviations (SDs) of Functional Independence Measure (FIM) and MMSE are shown in Table 1A (10). The number of patients in each stage of Brunnstrom is listed in Table 1B, but five patients' data were unavailable. Table 1C shows whether the lesion site is left, right, or both. Since the automated anatomical labeling (AAL) does not cover the brain stem, etc., there are no data on lesion degree in 11 patients, but it does not affect the results (7, 8). The severity of the patient is represented by the stage of Brunnstrom and FIM.

**Table 1:** Patient information.

**(A)**				
**Mean ±SD**	**FIM score**			**MMSE score**
	**Motor**	**Cognitive**	**Total**	
	69.7 ± 17.6	29.6 ± 5.3	98.7 ± 22.2	27.3 ± 2.5
**(B)**				
**Brunnstrom (patients number)**				
**Stage**	**Lower limbs**	**Upper limbs**	**Fingers**	
1–2	6	6	2	
3–4	13	13	12	
5–6	141	141	146	
**(C)**				
**Location of lesion (patients number)**				
**Hemisphere**			**Not applicable to AAL**	
**Right**	**Left**	**Both**		
64	41	49	11	

***(A)**, The mean and standard deviation of the subjects; **(B,C)** The number of subjects who meet each condition*.

The study was approved by the Ethics Committees of Shinaikai Hibino Hospital and the Hiroshima University Epidemiological Research Institute. All experiments were conducted in compliance with the relevant regulations and the latest version of the Declaration of Helsinki. Before we started any measurements, we obtained informed consent from either the patients or those authorized to provide consent on behalf of the patients, including family members or guardians.

### Imaging and Non-imaging Data

The T2-weighted axial brain-image data were obtained by 1.5-T MRI (Signa EXCITE, XI, version 11.0, GE Healthcare). The MRI imaging conditions are as follows: repetition time/echo time (4600/88 ms), duration (1 min 28 s), field of view (220 mm), matrix (320 × 256), slice gap (2 mm), and slice thickness (5 mm). To quantify lesions, the standard brain template of the Montreal Institute of Neuroscience (2 × 2 × 2 mm voxels) was used. Lesion sites were identified and manually labeled by a surgeon, using MRIcron software (11); two surgeons validated lesion sites independently and this method was guaranteed by two surgeons (κ-coefficient, 0.944) (12). The brain was divided into 116 regions that were classified by AAL (7, 8). For analysis, the number of voxels in each AAL area was calculated. Similarly, the number of voxels at lesions in each region was calculated. The number of voxels at lesions is divided by the number of voxels in each region, and the ratio was defined as the “lesion degree (%).”

Before TMT, the subjects were instructed how to do the test. When the subject reached the end of 25 points, the inspector recorded the completion time. If the subject could not perform during the test, the inspector recorded the points that they could reach. The point was defined as the score. In that case, there is no record of the completion time.

The imaging data and TMT-B data were collected during the subjects' hospitalization in the rehabilitation ward.

### Clustering and Significant Difference Testing

Although we had tried to find the differences in the lesion degree using the total data, a good model was not obtained. Therefore, we performed the subgroup analysis using the clustering method. Because using the final performance to determine the number of groups, *k* may result in overfitting, we used the elbow method that is not affected by the final performance (13). According to the results of the elbow method, data-driven and unsupervised *k*-means were performed only by score or only by time. Data with a completion time of 300 s or more were excluded. Since the hypotheses of normal distributions for the scores and completion times were rejected by the Shapiro–Wilk test, the Wilcoxon rank–sum test was used for significant difference testing between groups. Using the Benjamini–Hochberg (BH) method for multiple corrections of *p*-values, we calculated corrected *p-*values to identify brain sites with a significant difference (*p* < 0.1) (14). False positives among the multiple hypotheses were controlled by the BH method. The number of hypotheses was defined by the number of group combinations (e.g., group 1 *vs*. group 2; group 1 *vs*. group 3, etc.). For example, the comparison between four groups was associated with six hypotheses. The significances were confirmed by the corrected *p*-values (i.e., corrected *p* < 0.1), which were calculated as follows:


(1) $$\begin{array}{r} {corrected\;p}_{i}=\frac{p_{i}\times n}{i} \end{array}$$

where *i* is the rank of the *p*-value when sorted in ascending order among multiple hypotheses, *p*_*i*_ is the *p*-value of rank *i, n* is the number of hypotheses, and the corrected *p-*value indicates the respected *i* rank.

## Results

### Clustering Analysis by TMT-B Score and Completion Time for Patients With Stroke

**Figure 3:**
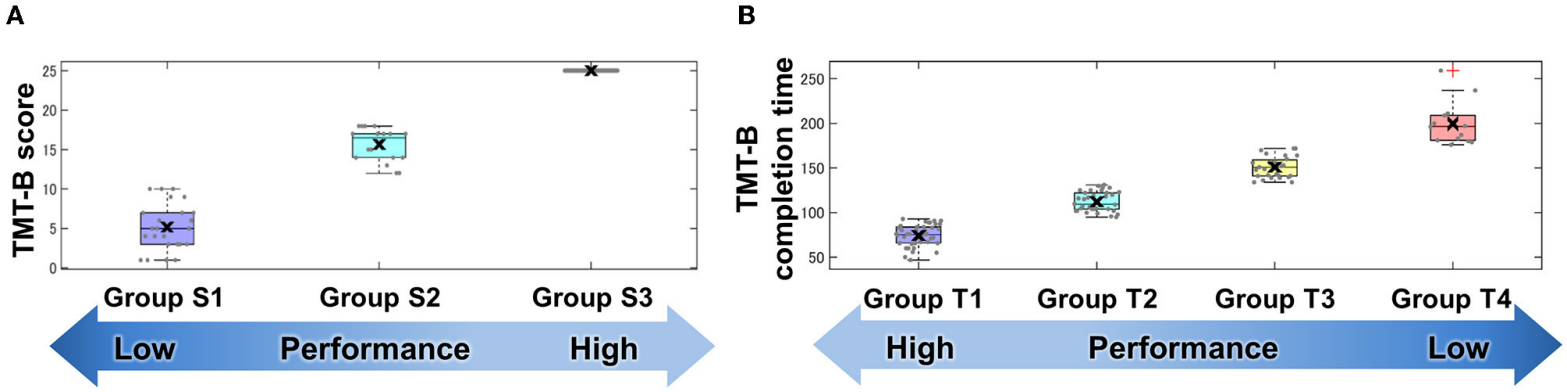
Groups classified by TMT-B score **(A)** and completion time **(B)**. Cluster analysis using TMT-B score and completion time. Data-driven unsupervised *k*-means clustering was performed to classify subjects into groups using **(A)** score and **(B)** completion time of TMT-B. Each point denotes subject-wise data. These results are shown in a box plot and the cross mark represents the median for each group.

The results of the classification by data-driven unsupervised *k*-means clustering are shown in Figure 3 and Table 2. Three groups, S1, S2, and S3, were obtained by classification based on the TMT-B score. The numbers of subjects in groups S1 (the low-score group, with a score ≤ 10), S2 (moderate, 12 < score ≤ 18), and S3 (high, score = 25) were 24, 18, and 123, respectively. As the typical clinical cut-off of TMT-B completion time is 300 s, 117 of the 122 subjects who reached 25 points within 300 s were classified into four groups, T1, T2, T3, and T4 based on their completion time. The numbers of subjects in groups T1 (47–93 s), T2 (95–131 s), T3 (134–172 s), and T4 (176–259 s) were 42, 34, 27, and 14, respectively.

**Table 2:** Groups classified by TMT-B score and completion time.

**Group**	**Group S1** **(low-score group)**	**Group S2** **(moderate-score group)**	**Group S3** **(high-score group)**	
Score	1–10	12–18	25	
Mean ± SD	5.2 ± 2.9	15.7 ± 2.0	25.0 ± 0.0	
Number of subjects	24	18	123	
**Group**	**Group T1**	**Group T2**	**Group T3**	**Group T4**
Completion time (s)	47–93	95–131	134–172	176–259
Mean ± SD	74.6 ± 11.6	112.4 ± 10.8	151.0 ± 11.6	199.5 ± 22.9
Number of subjects	42	34	27	14

### Analysis of Relationship Between Lesion Sites in Score Groups

**Figure 4:**
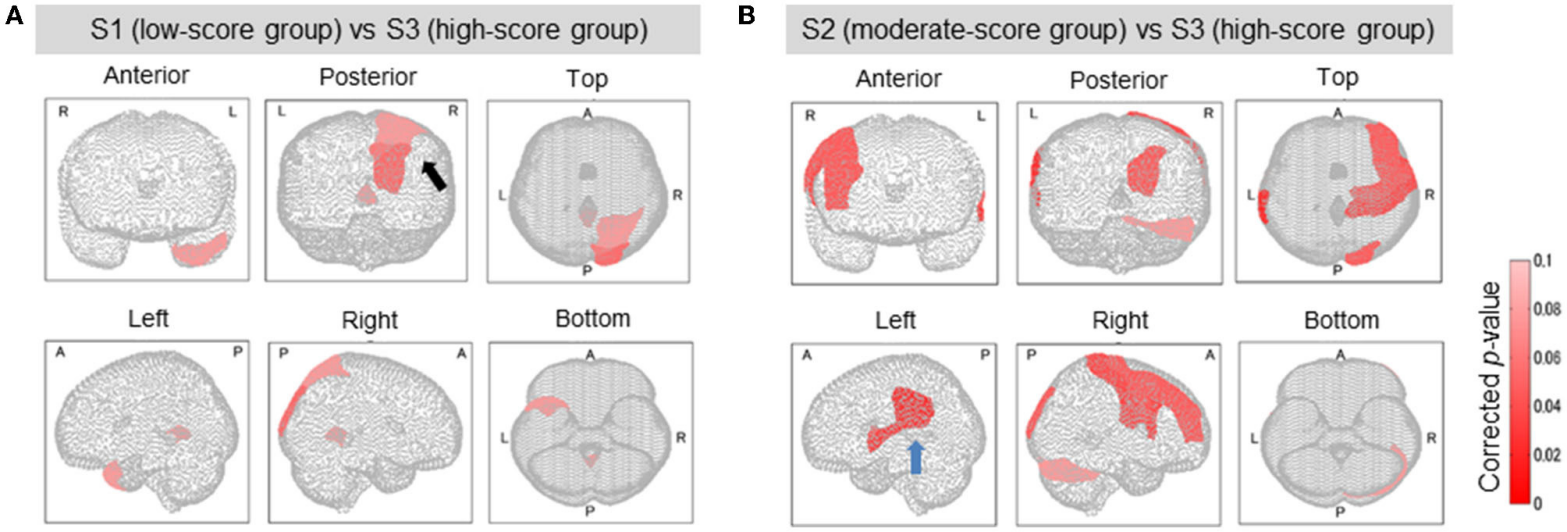
Brain regions with a significant difference in lesion degree according to TMT-B score. In the three groups classified by TMT-B score, the relationship between brain-lesion sites was verified by a Wilcoxon rank–sum test. Furthermore, the *p*-value was multiply-corrected by the BH method. In S1 (low-score group) and S3 (high), as shown in **(A)**, five brain regions showed a significant difference (corrected *p* < 0.1). In group S2 (moderate) and group S3 (high), as shown in **(B)**, 10 brain regions showed a significant difference (corrected *p* < 0.1). In (**A**, Posterior), the black arrow shows the cuneus and the superior occipital gyrus. In (**B**, Left), the blue arrow shows the supramarginal gyrus and the superior temporal gyrus.

**Figure 5:**
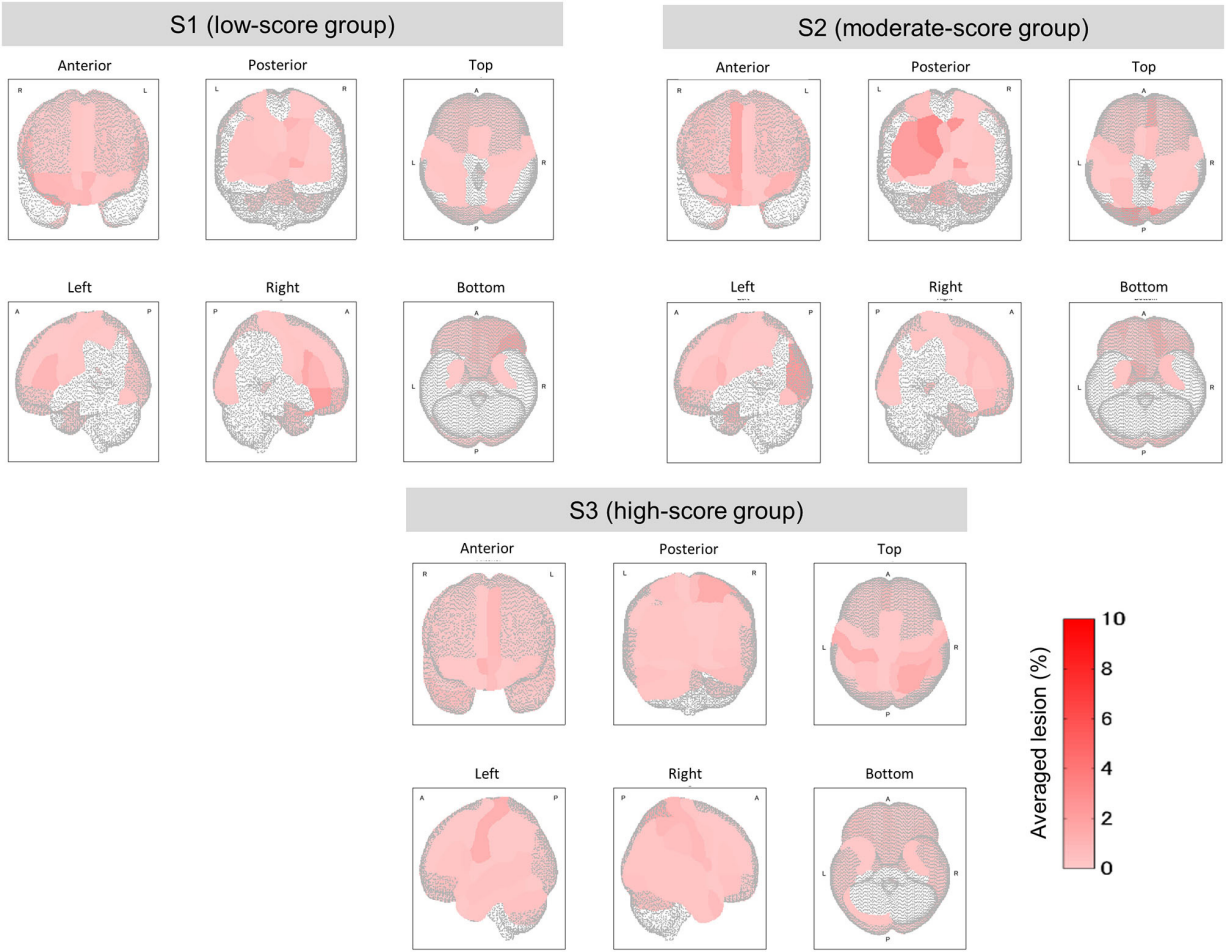
Mean lesion degree maps for three groups with low, moderate, and high TMT-B scores. In terms of TMT-B scores, the mean lesion degree at each site was calculated for comparison among groups. The brain sites with no lesion (the lesion degree is zero) are not colored.

The brain's lesion degree was calculated for each of the 116 regions classified by AAL. For each group, brain sites with significant differences (corrected *p* < 0.1) were evaluated and corrected as described in section Clustering and Significant Difference Testing. Figure 4 shows the brain sites with significant differences (corrected *p* < 0.1) between the two groups in red. For comparison between the groups, mean lesion degree maps for each group are shown in Figure 5.

The relationship between the lesion degree and the TMT-B score was determined by lesion analysis. As seen in Figure 4A, group effects were observed in several brain sites (Kruskal–Wallis *H* test; *p* < 0.1). The posterior lobe showed a particularly significant difference in the lesion degree. After BH, the *p*-value correction for multiple hypotheses was done in a region-wise manner; five prominent sites presented significant differences (corrected *p*-value < 0.1) between groups S1 (low-score group) and S3 (high) as tabulated in Table 3A.

**Table 3:** Lesion degree according to TMT-B score.

**(A)**						
**Brain site**	**Related function**	**Lesion degree (%)**				**Corrected** p^*^
		**Group S1** **(low-score group: 24 subjects)**		**Group S3** **(high-score group: 123 subjects)**		
		**Mean**	**Max**.	**Mean**	**Max**.	
Cuneus_R	Vision	0.099	2.317	0.001	0.070	0.053
Superior occipital gyrus_R	Vision	1.259	30.219	0.000	0.000	0.037
Superior parietal gyrus _R	Space attainability	0.053	1.260	0.000	0.000	0.074
Middle temporal pole _L	Emotion, cognitive processing	0.028	0.662	0.000	0.000	0.074
Vermis_4_5	Eye movement	0.100	2.406	0.000	0.000	0.074
**(B)**						
**Brain site**	**Related function**	**Lesion degree (%)**				**Corrected** p^*^
		**Group S2** **(moderate-score group: 18 subjects)**		**Group S3** **(high-score group: 123 subject)**		
		**Mean**	**Max**.	**Mean**	**Max**.	
Precentral gyrus_R	Motor hand	0.552	9.938	0.000	0.000	0.029
Middle frontal gyrus_R	Attention	0.029	0.529	0.000	0.000	0.029
Inferior frontal gyrus, opercular part_R	Language	0.500	8.435	0.035	4.289	0.015
Rolandic operculum _L	Language	0.011	0.202	0.000	0.000	0.029
Supramarginal gyrus _L	Language	0.407	7.166	0.000	0.000	0.001
Superior temporal gyrus _L	Language	0.765	13.240	0.001	0.131	0.014
Superior occipital gyrus _R	Vision	0.071	1.274	0.000	0.000	0.029
Postcentral gyrus _R	Somatic sense	0.357	6.356	0.004	0.523	0.015
Cerebellum_Crus1_R	Spatial navigation	0.520	7.440	0.046	4.985	0.07
Cerebellum_3_R	Motor	0.510	8.213	0.051	6.280	0.015

* *p-value was multiply-corrected by the BH method. Medians and minimums of lesion degree are 0.000 at all brain sites. In the table, L and R indicate “left” and “right,” respectively*.

Groups S2 (moderate) and S3 (high) were also subjected to the relationship analysis described above. According to these results, which are shown in Figure 4B and Table 3B, 10 brain sites showed significant differences in the lesion degree.

From the results of S1 *vs*. S2 and S2 *vs*. S3, the frontal and the temporal lobes were particularly affected. These results confirm that the TMT-B scores reflected the brain sites that were affected by lesions.

### Analysis of Relationship Between Lesion Sites in Completion Time Groups

**Figure 6:**
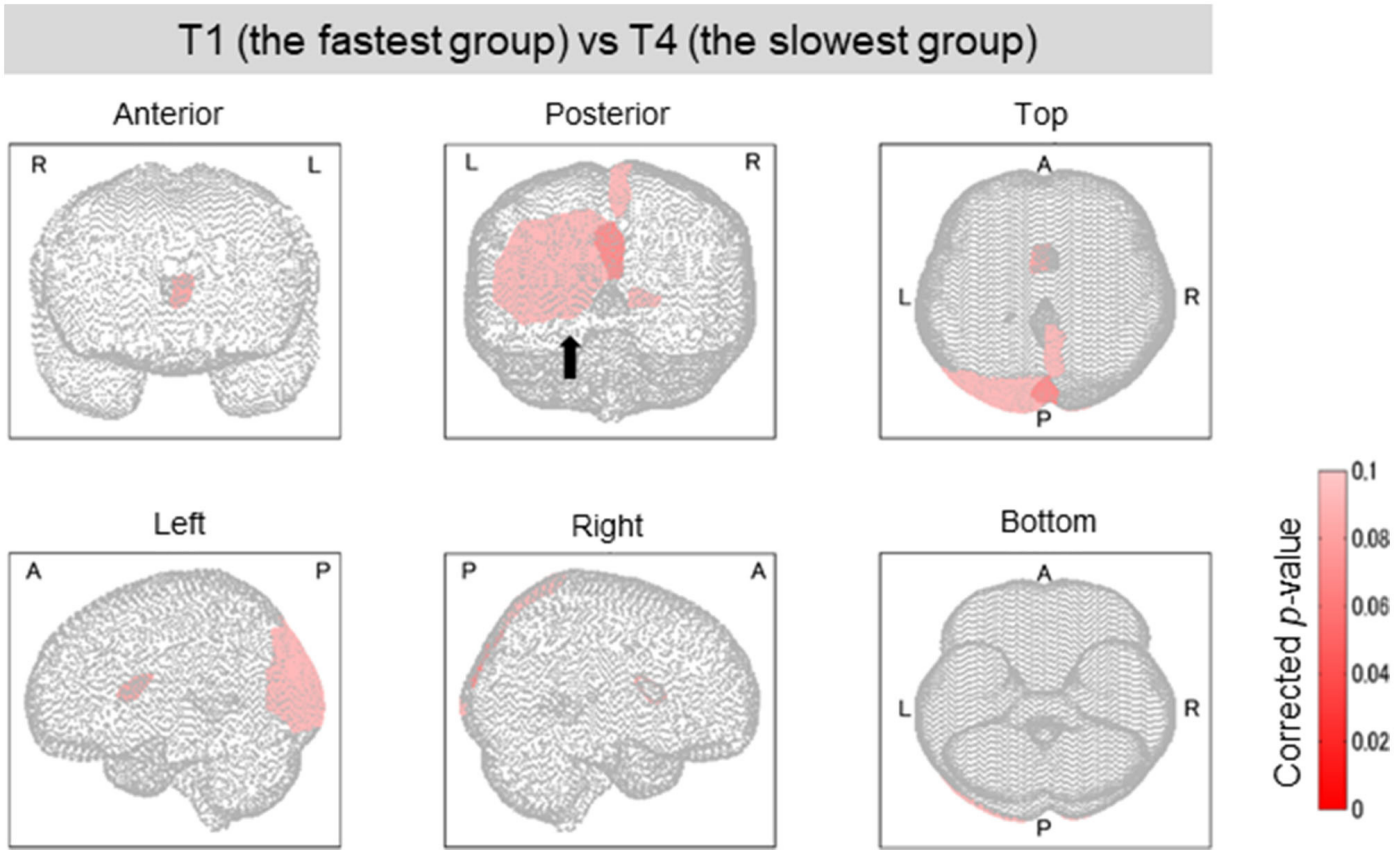
Brain regions with a significant difference in lesion degree according to TMT-B completion time. As for the four groups categorized by TMT-B completion time, the relationship between the brain-lesion sites was verified by a Wilcoxon rank–sum test. Further, the *p*-value was multiply-corrected by the BH method. Between groups T1 (fastest group) and T4 (slowest), six brain regions showed a significant difference (corrected *p* < 0.1). In Posterior, the black arrow shows the cuneus, the superior occipital gyrus, and the middle occipital gyrus.

**Figure 7:**
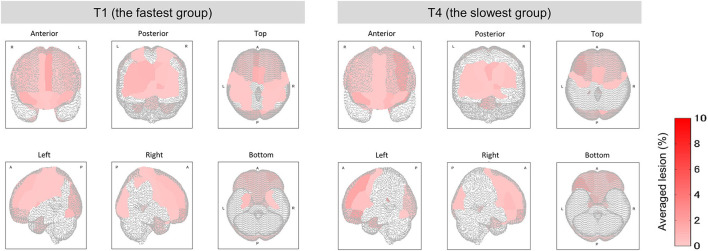
Mean lesion degree maps for the fastest and slowest groups to complete TMT-B. In terms of TMT-B completion time, the mean lesion degree at each site was calculated for comparison between groups T1 (fastest group) and T4 (slowest). The brain sites with no lesion (the lesion degree is zero) are not colored.

Among the four groups classified on the basis of their completion time, group T1 was characterized by the fastest completion times (<94 s) with slower times for groups T2 and T3, and the slowest times for group T4 (176 ≤ time ≤ 259 s). A higher function capacity is required to complete the TMT-B in a shorter time. We thus performed the lesion analysis to investigate the relationship between lesions and the TMT-B completion time. As shown in Figure 6, the group effects were observed in several brain regions (Kruskal–Wallis *H* test; *p* < 0.1). The posterior lobe and subcortex were particularly affected. After the correction for multiple hypotheses (BH correction; region-wise manner), six prominent regions presented significant differences (corrected *p*-value < 0.1) between groups T1 and T4, as tabulated in Table 4. The subjects in group T4 exhibited more severe lesion degrees in those regions. As information, mean lesion degree maps for groups T1 and T4 are shown in Figure 7.

**Table 4:** Lesion degree according to the completion time of TMT-B.

**Brain site**	**Related function**	**Lesion degree (%)**				**Corrected *p*** ^*^
		**Group T1** **(the fastest group: 42 subjects)**		**Group T4** **(the slowest group: 14 subjects)**		
		**Mean**	**Max**	**Mean**	**Max**	
Calcarine_R	Vision	0.003	0.107	0.203	1.666	0.094
Cuneus_L	Vision	0.033	1.376	0.239	2.294	0.059
Superior occipital gyrus _L	Vision	0.000	0.000	0.690	7.980	0.088
Middle occipital gyrus_L	Vision	0.000	0.000	0.389	5.352	0.088
Precuneus_R	Visuo-spatial imagery	0.000	0.000	0.350	4.839	0.088
Caudate nucleus _L	Spatial working memory	0.087	3.534	0.616	4.054	0.078

* *p-value was multiply-corrected by the BH method, and the corrected p-value was calculated. Medians and minimums of lesion degree are 0.000 at all brain sites. In the table, L and R indicate “left” and “right,” respectively*.

## Discussion

In terms of the TMT-B scores, five brain sites showed a significant difference in the lesion degrees between groups S1 (low-score group) and S3 (high). The cuneus and the superior occipital gyrus in the occipital lobe are part of the visual cortex (15, 16). The temporal lobe's temporal pole is reported to be related to emotion and cognitive processing (17). The parietal lobe's superior parietal gyrus has the function of space attainability, and the cerebellar vermis is related to eye movement and decreased cognitive function due to aging (18–20).

We found that the lesion sites in the low-score group were mainly responsible for vision and spatial attention. We also administered the TMT-A test, in addition to the TMT-B, 161 of 165 subjects obtained a perfect score of 25. Considering the TMT-A results, we can presume that the so-called vision disorder was not the reason for the low scores in group S1, although the visual cortex was damaged in those subjects. The visual cortex performs the processes of constructing an image from visual information and recognizing the image and its meaning. It is possible that impairment of letter recognition, i.e., the language function reduced the TMT-B scores.

Similarly, 10 brain sites showed a significant difference between group S2 (moderate-score group) and S3 (high). In the frontal lobe, the precentral gyrus is related to motor hand function, the middle frontal gyrus is related to attention, and the Rolandic operculum is related to language (21–23). The post-central gyrus of the temporal lobe is called the primary somatosensory cortex (24). The cerebellum is related to motor function in general, and the crus1 of the cerebellum is related to spatial navigation. The superior occipital gyrus is part of the visual cortex (15). On the left side of the temporal lobe, the supramarginal gyrus and the superior temporal gyrus, namely, Wernicke's area, are related to language processing (25, 26). In the inferior frontal gyrus, i.e., Broca's area has the role of producing language (27). Moreover, for language, the left side is said to be dominant, but the right side is also involved in speech (28). In the moderate-score group, it is a major feature that there was a significant difference in the site related to the language, such as the language center, Wernicke's area, and Broca's area. For the TMT-B, we presume that a high score could not be achieved because of the necessity to recognize letters. It is also thought that the attention function must be used more as the task complexity increases. As for the somatosensory system and motor function, because these subjects could perform the TMT-A, there was no apparent problem in the physical function. However, there is a network for the motor-language function, and that network might not have performed well (29).

Next, in terms of the TMT-B completion times, the functions of the brain sites where there was a significant difference between groups T1 (fastest group) and T4 (slowest) are as follows. In the occipital lobe, the calcarine, the cuneus, the superior occipital gyrus, and the middle occipital gyrus are visual areas (15, 30–32). The precuneus in the temporal lobe is related to visuo–spatial imagery, and the caudate nucleus is related to spatial working memory and learning (33–35). The common feature of the brain sites with a significant difference between groups T1 and T4 was thus the visual and spatial functions. Note that the TMT-B completion time refers to the time to connect all 25 points. Accordingly, because the subjects in group T4 had damage to the visual and spatial functions, it took them more time to recognize, memorize, and find the positions of the numbers and letters. As for group T1, the mean completion time was 74.6 ± 11.6 s. Although the compositions of age and sex are slightly different, the mean TMT-B completion times for healthy older people have been reported to be 81.5 ± 36.1 s (36). The results thus suggest that the T1 subjects had the same ability as healthy people. In other words, for the T1 subjects, the degree of the brain damage was light, or the lesion sites did not affect their performance of TMT-B.

**Figure 8:**
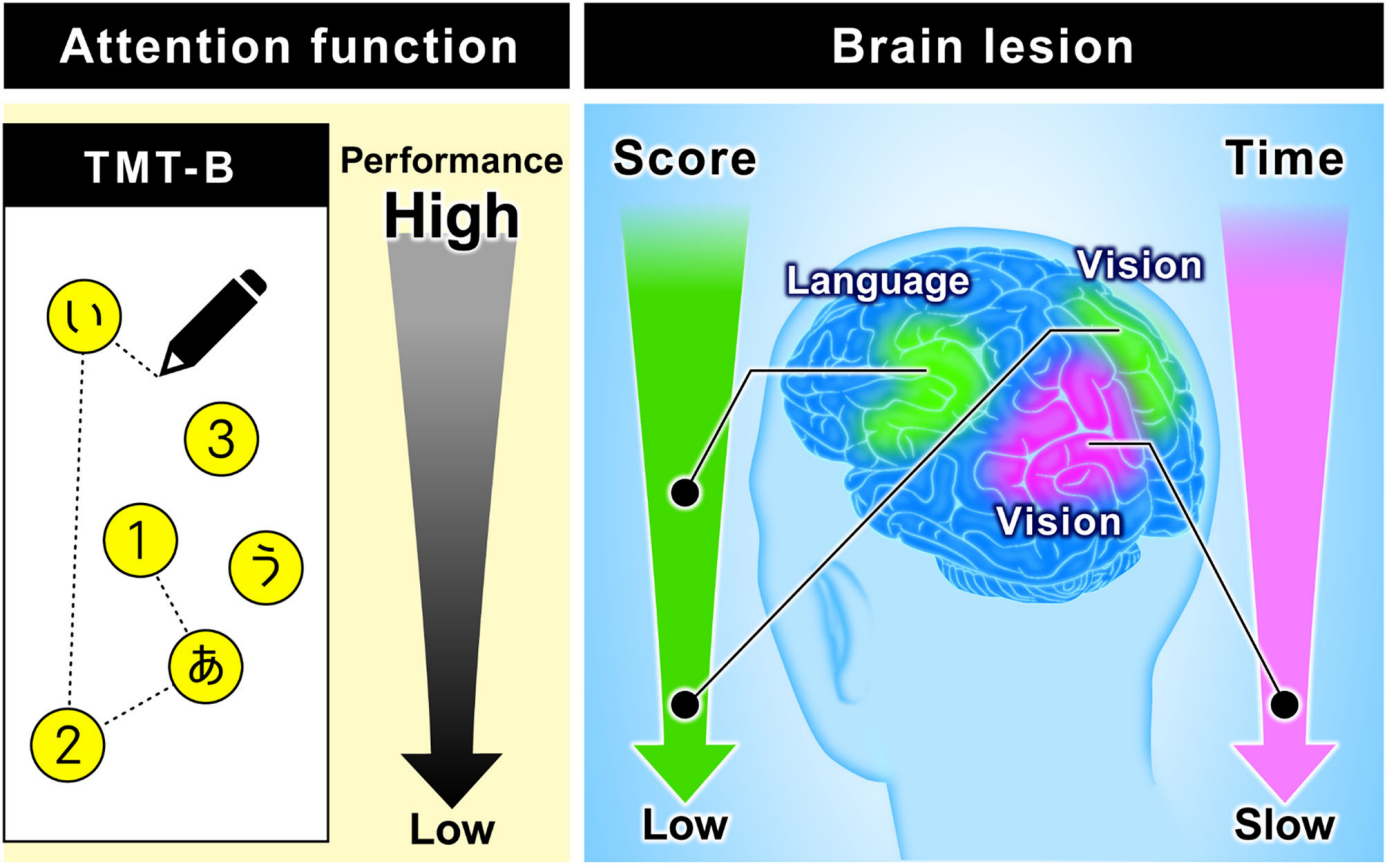
Relationship between TMT-B performance and brain function. The figure on the left is an image of TMT-B (Japanese version). As for TMT-B score, the subjects of the low-score group are mainly damaged in the visual area. On the other hand, the subjects of the moderate-score group are mainly damaged in the language area. As for TMT-B completion time, the subjects of the slowest group are mainly damaged in the visual area.

The TMT-B score is rarely used for evaluation, but stroke patients are often unable to reach 25 points. Accordingly, we added the score as an index in this study. As a result, we found a new relationship between the TMT-B result and the language area. Although this procedure reflects the characteristics of the TMT-B (using letters), the completion times to connect all 25 points showed no significant differences. By using the score and classifying the subjects in this manner, we showed that the subjects in the low-score group or the slowest group mainly had damage in the visual area, whereas the subjects in the moderate-score group mainly had damage in the language area. Figure 8 summarizes these results, which demonstrate the possibility of predicting the brain damage sites from TMT-B results.

In several previous studies (37, 38), it has been reported that the TMT-B completion time and frontal lobe are related. However, no significant difference in the frontal lobe has been found in this study. It is possible that the clustering in this study was not suitable for finding the relationship. Allen et al. refer to the brain sites that are activated during the TMT-B as visual pathways, the medial pre-supplementary area, and the premotor area (5). Similarly, in this study, the TMT-B results indicate that brain sites with damaged visual and motor areas resulted in low scores and longer completion times. In the study of healthy subjects, the sites where TMT-A and B differ are the left middle temporal gyrus and superior temporal gyrus (39). These sites were also detected in our study.

### Limitations

We used the data of the subjects who can understand the instructions and carry out the tests including TMT, MMSE, and other higher brain-function tests, excluding the subjects with severe aphasia that cannot perform tests. The patients with a low score in MMSE (<20) were also excluded because of the higher aphasia risk. As for hemi-lateral spatial neglect, we think that the effect would have been small because most subjects completed the TMT-A. Although we found a relationship between TMT-B results and the language and visual areas, we also plan to perform language and visual function tests and compare those results with the results of this study.

Each AAL area does not have the same volume. If the volume of an area is large; therefore, the possibility that the area has a lesion is increased. Moreover, in this study, there are more patients with lesions on the right side than those with lesions on the left side. In each AAL area, the number of patients with lesions within it varies. Accordingly, more data are needed for further generalization.

The results of subgroup analysis depend on the number of clusters depending on the dataset. As arbitrary processes were excluded, a series of analyses in this study are applicable to an expanded dataset. More data will provide more accurate results.

## Data Availability Statement

The raw data supporting the conclusions of this article will be made available by the authors, without undue reservation.

## Ethics Statement

The studies involving human participants were reviewed and approved by the Ethics Committee of Shinaikai Hibino Hospital and the Hiroshima University Epidemiological Research Institute. The patients/participants provided their written informed consent to participate in this study.

## Author Contributions

SH and KS performed the measurements for subjects. KS carried out the lesion analysis by labeling the lesion location. HA performed the quantitative analysis of lesion degree. AN and SS conceived the idea and developed the remaining analysis method. MK, HA, AO, TF, TM, and TT verified the analysis method. AK, SH, and TT supervised this study. All authors discussed the results and contributed to the final manuscript. All authors contributed to the article and approved the submitted version.

## Conflict of Interest

AN, SS, MK, HA, AO, TF, and AK were employed by Hitachi Ltd. TM was employed by Maxell Ltd. The remaining authors declare that the research was conducted in the absence of any commercial or financial relationships that could be construed as a potential conflict of interest.

## Publisher's Note

All claims expressed in this article are solely those of the authors and do not necessarily represent those of their affiliated organizations, or those of the publisher, the editors and the reviewers. Any product that may be evaluated in this article, or claim that may be made by its manufacturer, is not guaranteed or endorsed by the publisher.
